# Human recombinant RNASET2: A potential anti-cancer drug

**DOI:** 10.18632/oncoscience.295

**Published:** 2016-03-04

**Authors:** Levava Roiz, Patricia Smirnoff, Iris Lewin, Oded Shoseyov, Betty Schwartz

**Affiliations:** ^1^ T2 BIOTECH Ltd, Weizmann Science Park, Ness Ziona, ISRAEL; ^2^ The Robert H. Smith Institute of Plant Science and Genetics in Agriculture and School of Nutritional Sciences Institute of Biochemistry, Food Science and Nutrition, The Robert H. Smith Faculty of Agriculture, Food and Environment, The Hebrew University of Jerusalem, Rehovot, ISRAEL; ^3^ School of Nutritional Sciences Institute of Biochemistry, Food Science and Nutrition, The Robert H. Smith Faculty of Agriculture, Food and Environment, The Hebrew University of Jerusalem, Rehovot, ISRAEL

**Keywords:** actin-binding, tumorigenesis, angiogenesis, ribonuclease

## Abstract

The roles of cell motility and angiogenetic processes in metastatic spread and tumor aggressiveness are well established and must be simultaneously targeted to maximize antitumor drug potency. This work evaluated the antitumorigenic capacities of human recombinant RNASET2 (hrRNASET2), a homologue of the *Aspergillus niger* T2RNase ACTIBIND, which has been shown to display both antitumorigenic and antiangiogenic activities. hrRNASET2 disrupted intracellular actin filament and actin-rich extracellular extrusion organization in both CT29 colon cancer and A375SM melanoma cells and induced a significant dose-dependent inhibition of A375SM cell migration. hrRNASET2 also induced full arrest of angiogenin-induced tube formation and brought to a three-fold lower relative HT29 colorectal and A375SM melanoma tumor volume, when compared to Avastin-treated animals. In parallel, mean blood vessel counts were 36.9% lower in hrRNASET2-vs. Avastin-treated mice and survival rates of hrRNASET2-treated mice were 50% at 73 days post-treatment, while the median survival time for untreated animals was 22 days. Moreover, a 60-day hrRNASET2 treatment period reduced mean A375SM lung metastasis foci counts by three-fold when compared to untreated animals. Taken together, the combined antiangiogenic and antitumorigenic capacities of hrRNASET2, seemingly arising from its direct interaction with intercellular and extracellular matrices, render it an attractive anticancer therapy candidate.

## INTRODUCTION

Cell motility is a key prerequisite for cancer cell invasiveness and metastatic spread, and has become the focus of many research efforts aiming to identify the mechanisms empowering cell transformation from a stationary to migratory state. Regulation of cell migration is projected to bear significant potential in effectively treating cancer or preventing its progression. Actin cytoskeleton reorganization has been pinned as a key element underlying cell motility and invasion [1] and therefore presents an attractive target in antitumorigenic and antimetastatic agent design. In parallel, tumor progression and metastasis are intimately associated with angiogenic processes, which ensure both nutrient and oxygen supply to the rapidly growing tumor and access to a route for spread from the original tumor site. For many tumor types, relapse and poor prognosis correlate with elevated circulating levels of angiogenic factors and overall metastatic potential and patient outcome can be predicted by microvessel density [2, 3]. More specifically, increased serum concentrations of angiogenin, VEGF, bFGF and IL-8 [4] positively correlate with disease stage, tumor burden and overall survival rates. Various melanomas, in particular, feature distinct angiogenic processes, which have been shown to rapidly enhance their aggressiveness and progression [5]. Thus, many therapeutic approaches aim to achieve tumor regression or dormancy by targeting angiogenic factors and regulators. However, melanoma progression has been observed in vessel-dense organs exposed to antiangiogenic therapy, a phenomenon ascribed to co-option of existing vessels [3, 6]. Thus, combined treatments or administration of agents that target multiple pathways underlying tumor progression will inevitably display enhanced antitumor potency.

Apart from their well established function in RNA processing, ribonucleases (RNAses) have been implicated in a range of biological processes. Human angiogenin and eosinophil cationic protein, two ribonuclease-A superfamily members, display angiogenic and cytostatic capacities, respectively [7, 8], while RNase I correlated with a tomato cell nutritional stress-induced rescue mechanism [9]. In contrast, dimerized seminal bovine RNase reportedly exhibits cytosolic RNase inhibitor evasiveness and imparts significant cytotoxicity [10]. Similarly, amphibian oocyte-derived onconase effectively inhibited proliferation and reduced viability of exponentially growing cell lines *in vitro* and complete vascular destruction in FSaII tail tumors and growth of murine mammary adenocarcinoma and fibrosarcoma tumors *in vivo* [11]. ACTIBIND, an *Aspergillus niger* T2RNase family member, directly binds cell surface actin and crosslinks F-actin filaments arresting pollen tube elongation and disrupting their orientation [12, 13]. Similarly, it has been reported to trigger dose-dependent inhibition of both angiogenin-and bFGF-induced HUVEC tube formation, significantly attenuate the colony-forming capacities of various cancerous human cell lines [13] and disrupt intracellular actin networks and motility of cancerous cell lines. Moreover, development of both mouse and rat xenograft tumors was inhibited by ACTIBIND [13, 14], by its direct competition with angiogenin. Furthermore, subcutaneously delivered melanoma cells failed to form palpable tumors in ACTIBIND-treated mice within the time span they formed tumors in control mice; on day 16 of the study, melanoma tumors in ACTINBIND-treated mice were >10-fold smaller than their control counterparts. In addition, the incidence and counts of lung metastases were significantly lower in ACTIBIND-treated mice, which corroborated with the reduced matrix metalloprotein 2 (MMP2) expression and activity recorded in both melanoma and human umbilical vascular endothelial cells (HUVEC) [14]. The observed depletion of actin-rich cell extensions and rearrangement of cytoplasmic actin filaments upon exposure to ACTIBIND, further implicate its direct impact on cell motility [13]. Indeed, invasiveness of ACTIBIND-treated HT-29 and ZR-75-1 cells through Matrigel was blunted in a dose-dependent manner, reaching 80% inhibition in the presence of 10 μM ACTIBIND. Its potent antiangiogenic and antitumorogenic properties have been shown to be independent of its RNA hydrolyzing function [13].

The ubiquitous T_2_-RNase family members are evolutionarily conserved with representatives in viruses, mammals and plants, where deletion of the chromosomal region housing the ACTIBIND human homologue RNASET2 gene is associated with several human malignancies [15-18]. The recombinant human RNASET2 (hrRNASET2) demonstrated actin-binding capacities and curbed HT-29 clonogenicity, independent of its ribonuclease activity [19]. Much like its fungal counterpart, it inhibited angiogenin-, bFGF-and VEGF-induced tube formation and significantly inhibited LS174T tumor formation in athymic mice [19]. The current work set out to further characterize the antitumorigenic capacities of hrRNASET2 expressed in and isolated from Chinese hamster cells (CHO), in efforts to better define its potential as an anticancer drug. hrRNASET2 disrupted the organization of intracellular actin filaments and of the actin-rich extracellular extrusions in both CT29 colon cancer and A375SM melanoma cells. In addition, it significantly inhibited A375SM cell migration through Matrigel. Moreover, hrRNASET2 inhibited both tumor progression and metastatic spread in colorectal and melanoma tumor models, and proved to be more effective than the gold-standard anti-angiogenic drug Avastin. Thus, the combined antiangiogenic and antitumorigenic capacities of hrRNASET2 render it an attractive robust candidate for anticancer therapies.

## RESULTS

### Expression and characterization of hrRNASET2

**Figure 1:**
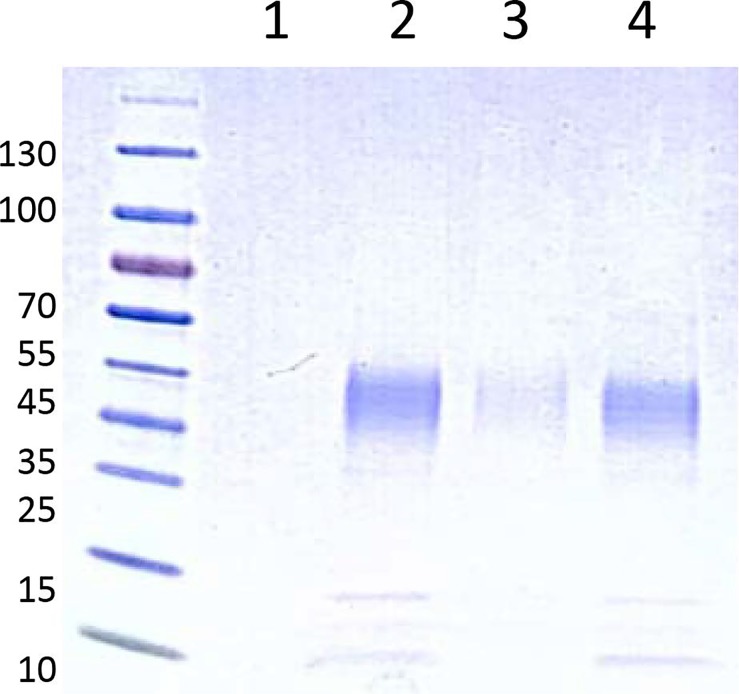
SDS-PAGE analysis of hrRNASET2 Recombinant human RNASET2 was expressed in CHO cells, affinity purified on a Ni sepharose column and then separated by SDS-PAGE, blotted and detected with mouse anti-hrRNASET2 antibodies (12.5%). Lane 1 – MW marker; lanes 2,3,4 – three different batches of hrRNASET2. The band is in the range between ∼ 34-45 kDa indicating the size of the glycosylated protein.

**Figure 2:**
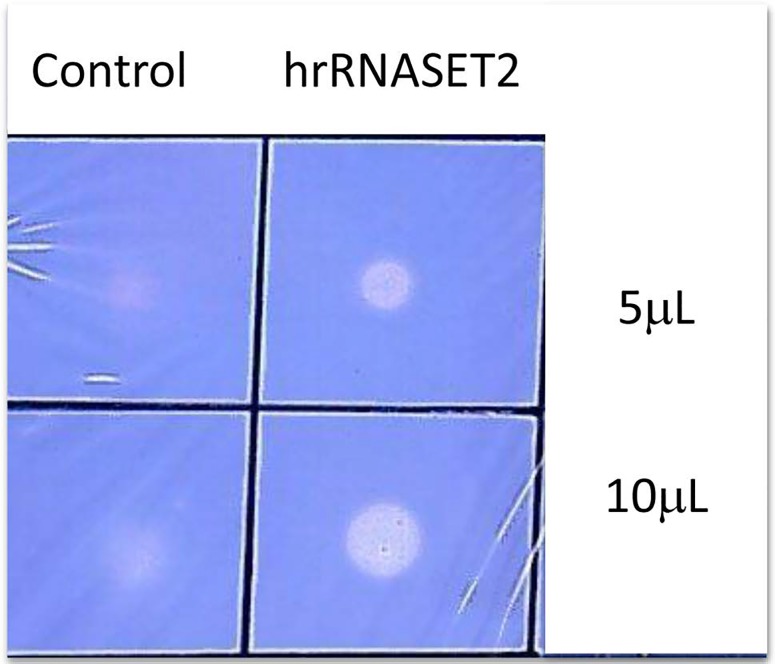
RNase activity assay The RNase activity of purified hrRNASET2 was assayed by observing the change in fluorescence intensity of toluidine blue on binding with yeast RNA. hrRNASET2 samples (10 μl) were placed over a plate containing 10 ml 0.1% yeast RNA and 0.8% agarose, and incubated for 30 min (37°C). Agarose plates were stained with 0.02% (w/v) toluidine blue to detect RNase activity. RNase activity was quantified by placing samples (50 μl) in 50 μl 100 mM sodium acetate buffer (pH 4.5) that contained 4 mg/ml yeast RNA.

The recombinant human RNASET2 protein was expressed in CHO cells and affinity-purified on a Ni sepharose column, yielding 90-100 mg purified freeze-dried hrRNASET2 per 24 liters CHO-K1-conditioned medium. The 45 kDa glycosylated hrRNASET2 protein detected on Coomassie-stained SDS PAGE (Figure 1) and Western blot (not shown), demonstrated ribonucleolytic activity against yeast RNA, as determined by standard zymography (Figure 2).

**Figure 3:**
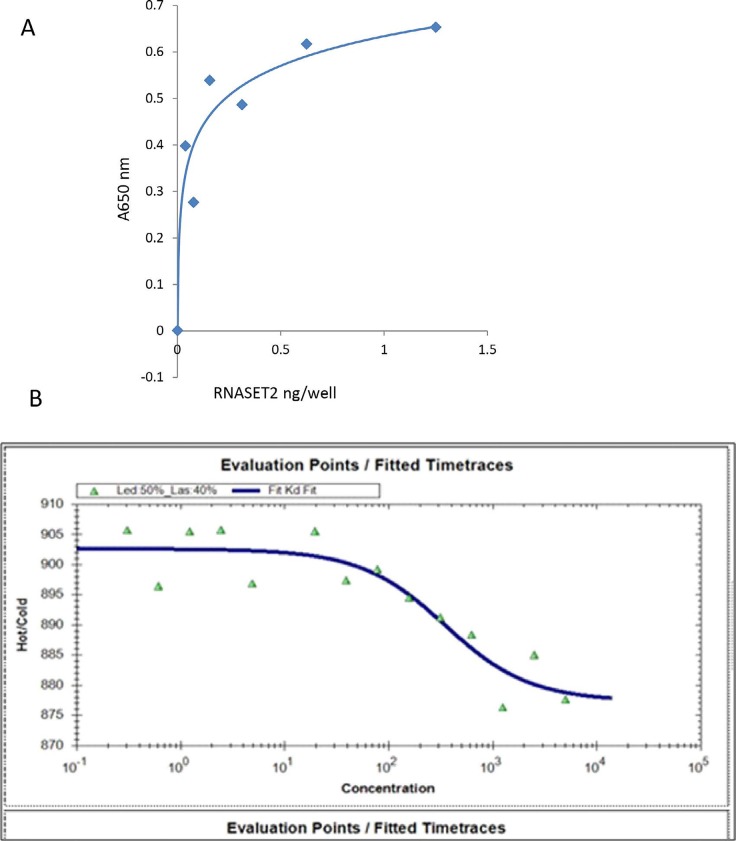
Actin binding capacity of hrRNASET2 **A.** Indirect ELISA to assess binding capacity of hRNASET2. Serial dilutions of hrRNASET2 were incubated in actin-coated wells (ON, 4°C), which were then washed and exposed to rabbit anti-RNASET2, followed by peroxidase-conjugated goat anti-rabbit IgG. Antibody binding was then quantified using TMB-ELISA solution and measurement of optical absorbance at 650 nm. **B.** Quantification of the equilibrium binding constant (KD=91.5nM) of actin and hrRNASET2 by MST. Serial dilutions of hrRNASET2 were incubated with fluorescently labeled actin (RT, 5 min) before being loaded into MST glass capillaries for analysis.

hrRNASET2 bound immobilized actin in a dose-dependent manner, reaching saturation at 0.2 ng/well (Figure 3A). Microscale thermophoresis (MST) analysis assessing hrRNASET2 actin binding capacities under close to native conditions, measured a dissociation constant of 91.5 nM (Figure 3B).

### hrRNASET2 curbs cell migration and angiogenesis

**Figure 4:**
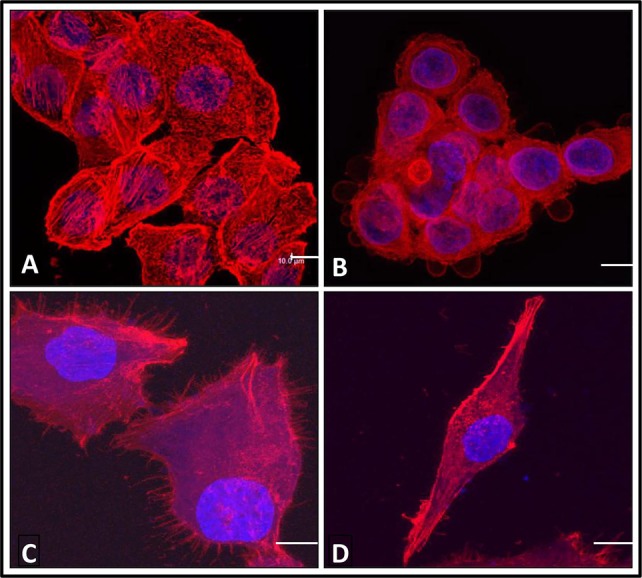
The effect of hrRNASET2 on cancer cell morphology Confocal microscopic analysis of HT-29 colon cancer cells **B.** and A375SM melanoma cells **D.** exposed to hrRNASET2 (1 mM) for durations of time and then stained with Rhodamine-Phalloidin for F-actin (red). Counterstain – DAPI (Nuclei – blue). Control cells were left untreated **A,C.** (Scalebar=10um).

**Figure 5:**
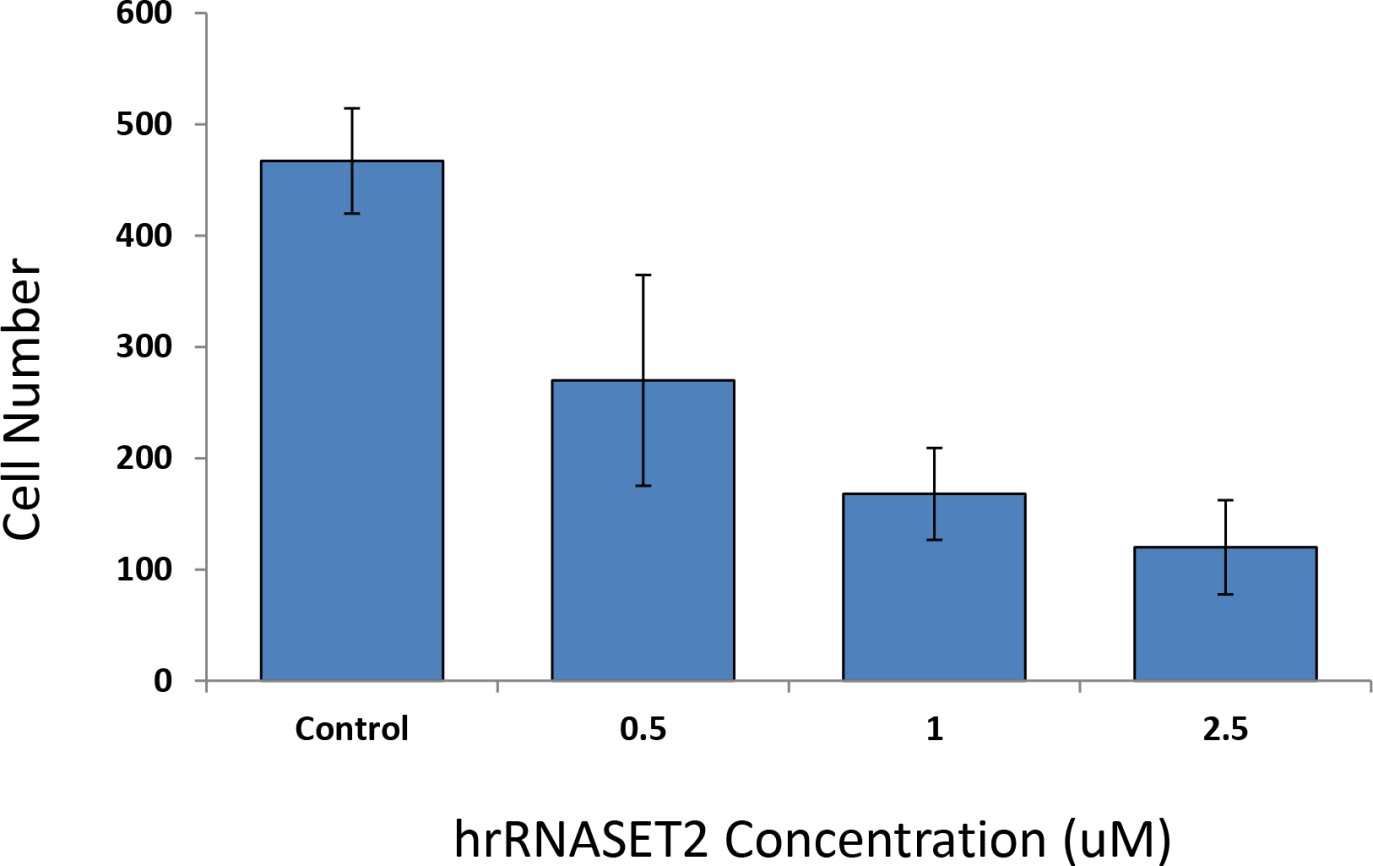
hrRNASET2 dose-dependent inhibition of cell migration A375SM cells (5×10^3^ cells/ml) suspended in serum-free medium supplemented with hrRNASET2 (2.5, 1.0 or 0.5 μM) or PBS< were seeded in the upper compartment of BioCoat™ Matrigel Invasion Chambers. The lower compartment contained NIH 3T3 fibroblast-conditioned medium, which served as a chemoattractant. Following incutation (37°C, 24 h), the membranes were stained with Diff-Quick solution and cells were counted under a microscope (x10 magnification). The data are expressed as the average number of cells counted in 10 fields in each of the three experiments ± SD.

The biological activity of hrRNASET2 was assessed in a series of assays, which focused on its reported impact on the cytoskeleton, cell migration, and angiogenesis. Immunostaining analysis of hrRNASET2-treated CT29 colon cancer cells demonstrated a rounded morphology, the appearance of membrane blebs, disruption of the intracellular actin filaments network and distinct accumulation of actin along the periphery of the cell (Figure 4 A,B). Similarly, A375SM human melanoma cells exposed to hrRNASET2 were less spread out than their untreated counterparts and fully lacked extracellular actin-rich extensions (Figure 4 C,D). In line with these marked alterations in intracellular and extracellular actin configurations a 24-hour incubation period with hrRNASET2 significantly impaired cell migratory capacities in a dose-dependent manner, with up to 74% fewer A357SM cells successfully traversing the Matrigel, when compared to untreated controls (Figure 5). Extension of the incubation time with hrRNASET2 to up to 94 hours resulted in 94% inhibition of motility (data not shown).

**Figure 6:**
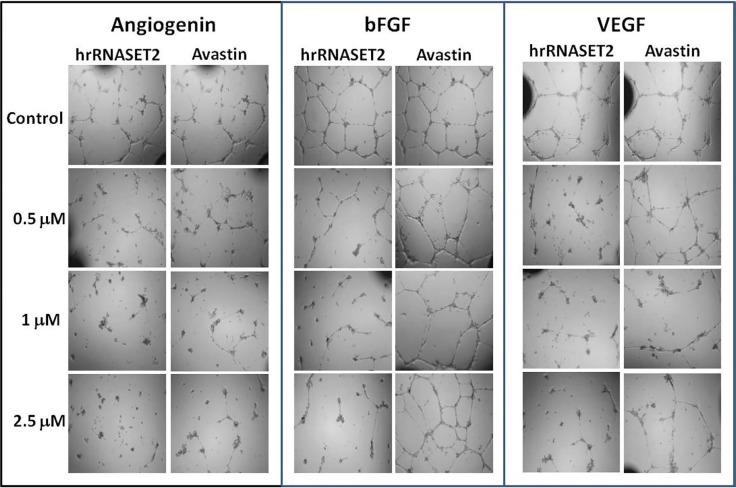
hrRNASET2-driven inhibition of HUVEC tube formation HUVEC were plated in growth medium supplemented with angiogenin, bFGF or VEGF (1 μg/ml each). hrRNASET2 (2 μM final concentration) or Avastin (n= 5 for each treatment) was then added. Cells were incubated overnight before tube formation was assessed.

In addition, much like hrRNASET2 expressed in *P. pastoris*, dose-dependent pollen tube growth inhibition was observed upon incubation of Lilly pollen with CHO cell-derived hrRNASET2, with a maximal effect of 47% growth inhibition measured for >1mM hrRNASET2 (Supplementary Figure S2). Similarly, the protein demonstrated a potent antiangiogenic effect at doses as low as 0.5 μM, as manifested by full arrest of angiogenin-induced HUVEC tube formation (Figure 5). A similar antiangiogenic effect was observed in both bFGF-and VEGF-induced HUVECs (Figure 6). hrRNASET2 demonstrated a more potent antiangiogenic effect than an identical dose of Avastin, which enabled a significant degree of angiogenesis (Figure 6).

### hrRNASET2 inhibits tumor growth, metastatic spread and prolongs animal survival

**Figure 7:**
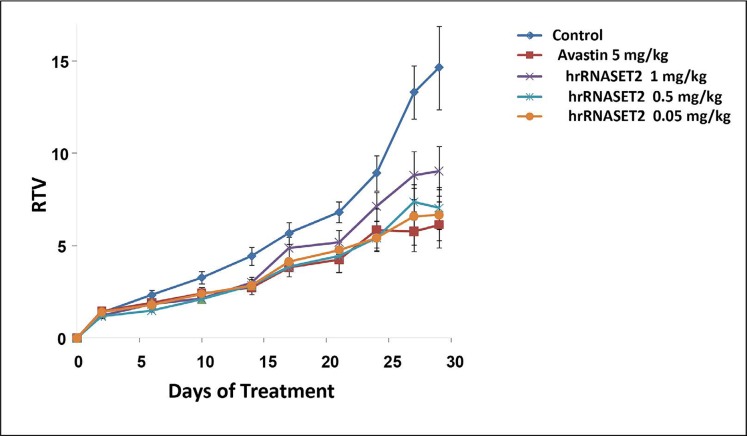
Inhibition of HT-29 colon cancer cells in a xenograft mouse model HT-29 cells were injected s.c. into the left hip of the mouse (106 cells/mouse). When the tumor diameter reached ∼100 mm^3^, the mice (n=6) were distributed randomly to either be treated i.v. every other day for 30 days with PBS (control) or with different doses of hrRNASET2. Avastin (5mg/kg) was injected twice a week for 30d, as a positive control.

**Figure 8:**
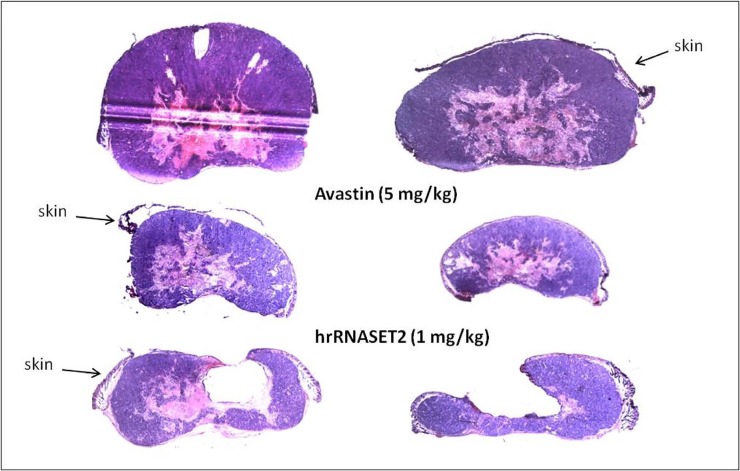
hrRNASET2-driven inhibition of tumor growth Cross-sections of tumors collected 30 days after the initial treatment, were stained with H&E. The blue-purple area represents viable cancer cells, where the pink area represents necrotic/apoptotic tissues. The tumors presented here represent the average size of tumors of each treatment.

**Figure 9:**
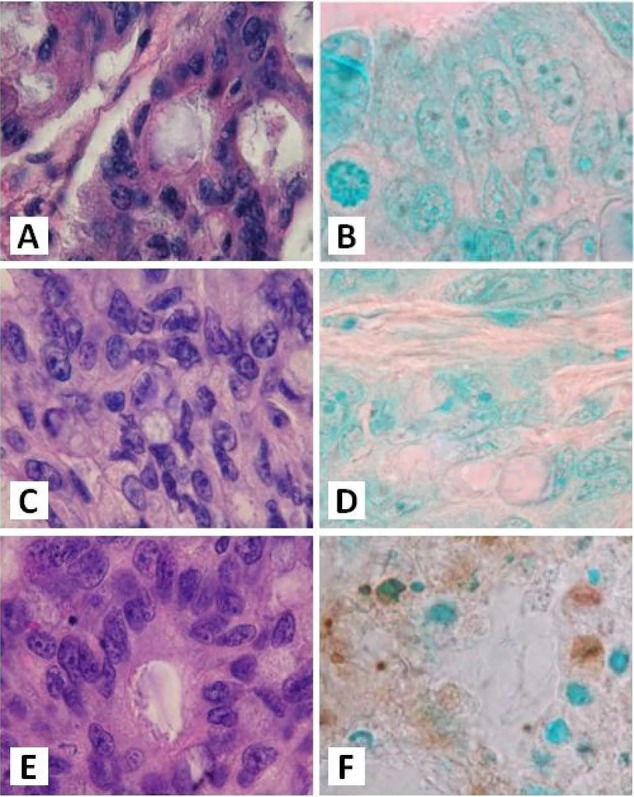
The effect of hrRNASET2 on tumor cell apoptosis Cross-sections of tumors collected 30 days after the initial treatment: **A,B.** – control; **C,D.** – Avastin-treated tumors; **E,F.** - hrRNASET2 - treated tumors. A,C,E - H&E staining. B,D,F – apoptosis TUNEL assay.

The antitumorigenic potential of hrRNASET2 was evaluated in subcutaneous HT29 cell tumor-bearing athymic mice intravenously treated with varying doses of hrRNASET2, every other day over a 30-day period. All hrRNASET2-treated animals presented significantly smaller tumors when compared to untreated controls (p<0.05), with the largest effect of an up to 65% tumor size reduction obtained with the 0.05 mg/kg hrRNASET2 regimen. No significant differences in tumor size measurements were observed between the various hrRNASET2 treatment groups. In addition, despite the ≥5-fold difference in administered doses, all tested hrRNASET2 regimens proved as effective as Avastin, and starting on day 10, maintained the relative tumor volume (RTV) significantly lower than that of the control RTV. This difference peaked by the end of the experiment on day 30, when the RTV of RNASET2-treated tumors was 3-fold lower than the control RTV (Figure 7). Histological analysis of tumors extracted from hrRNASET2-treated animals on day 30 of the study, revealed fewer viable cells, situated at a distance from blood vessels (Figure 9E), and huge cavities within the spongy tumor mass as compared to untreated control (Figure 8). Following hrRNASET2 treatment, mean blood vessel counts were significantly lower than those of control animals or Avastin-treated animals, plummeting to 30.7% and 36.9% of counts in control and Avastin-treated animals, for the 0.05 mg/kg hrRNASET2 dose (Table 1). Vessel area was significantly smaller among all treated animals, ranging between 1.9-2.7-fold lower than in the control group (Table 1). In parallel, widespread apoptosis was apparent (Figure 9F), especially around blood vessels. Tumors exposed to Avastin treatment were also smaller and less viable, but displayed neither lesions of any size (Figure 8), nor detectable apoptotic foci (Figure 9D) similarly to control samples (Figure 9B). Moreover, as in control samples, blood vessels endothelial cells appeared intact and were in close contact with viable tumor cells (Figure 9A,C).

**Table 1:** Inhibition of neovascularization

Treatment	Blood vessels ± SE	Relative Area of Blood Vessels ± SE %
Control-PBS	44±381^a^	0.7±9.7^a^
Avastin (5mg/kg x2/week)	43±317^a^	0.9±5.1^b^
hrRNASET2 (1mg/kg x3/week)	38±134^b^	0.6±3.6^b^
hrRNASET2 (0.5 mg/kg x3/week)	21±118^b^	0.8±4.0^b^
hrRNASET2 (0.05mg/kg x3/week)	14±117^b^	1.3±5.2^b^

Different letters indicate a significant difference at *P*<0.01.

n=10 microscopic fields for each treatment.

**Figure 10:**
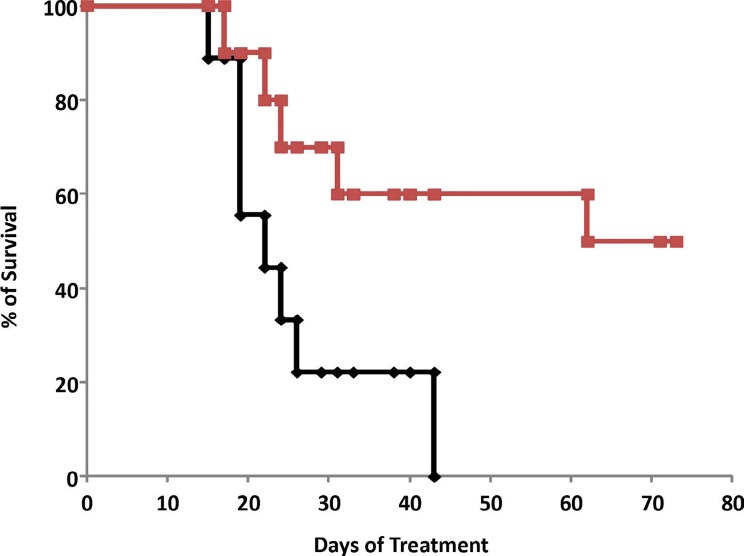
Elevated survival in hrRNASET2-treated A375SM-melanoma bearing mice Mice were injected s.c. with A375SM melanoma cells (2.5×105 cells/mouse). When the tumors reached ∼100 mm^3^, the mice were injected i.v. with hrRNASET2 (1mg/kg, n=10) or PBS (Control, n=9) every other day. The experiment was ended after 73 days. Survival curves were estimated by the Kaplan-Meier method. The log-rank test was performed to assess the significance of differences in survival curves.

The subcutaneous A375SM xenograft model yielded similar results, with hrRNASET2-treated animals displaying significantly improved survival rates when compared to untreated animals. By the close of the study on day 73 from initial treatment, 50% of the hrRNASET2-treated mice were alive and viable (Figure 10), whereas the median survival time for untreated animals was 22 days. Moreover, a number of the surviving animals demonstrated very small tumors (tumors had almost fully vanished) on day 73, leaving behind a scar in the original location of the tumor.

**Figure 11:**
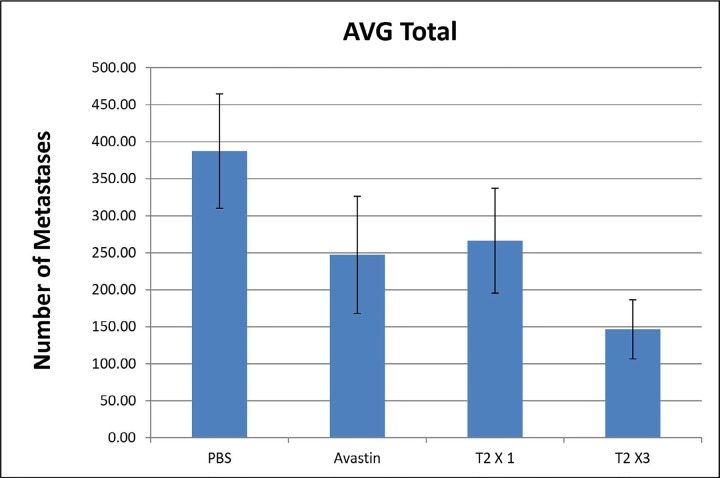
hrRNASET2-driven inhibition of A375SM melanoma lung metastases A375SM cells were inoculated into the tail vein of female nude mice, who were then intravenously treated for 60 days with: PBS as control, Avastin (2.5 mg/kg) × 2/week as positive control, hrRNASET2 (1 mg/kg) × 1/week (T2×1) or hrRNASET2 (1 mg/kg) × 3/week (T2×3). The lungs were excised and fixed and the macroscopic tumor nodules were counted under a dissecting microscope.

Upon i.v. injection of A375SM melanoma cells into the tail vein of nude mice, followed by various 60-day hrRNASET2 treatment regimens, a 2.6-fold reduction in mean lung metastasis counts was recorded among animals treated on a three times per week hrRNASET2 regimen, in comparison to untreated animals (see Table 2). The once per week hrRNASET2 regimen was less effective but provided protection from metastatic spread equivalent to that provided by Avastin (Figure 11 and Table 2).

**Table 2:** Inhibition of Metastasis (number of metastases per lung in each treatment (n=9-10)

Treatment	Average ± SE	Median	Range
Control-PBS	387±77	407.4	634 - 31
Avastin x2	247±79	278.5	587 - 10
hrRNASET2 x1	266.3±71	297.2	593 - 10
hrRNASET2 x3	146.5±40	142.8	350 - 6

## DISCUSSION

The present study demonstrates the multifunctional role of hrRNASET2 in numerous processes integral to tumor survival and spread, rendering it a unique agent with a projected high therapeutic potential. Its inhibitory impact on angiogenesis and metastatic spread has been related to its interaction with both intracellular actin cytoskeletal elements and peripheral actin-rich extracellular extensions. These observations are believed to underlie its significant dose-dependent inhibitory effect on cell motility as well as the protein's marked impact on both angiogenin-triggered HUVEC tube formation and pollen tube growth. These effects were further manifested in tumor xenograft models of the aggressive human A375SM melanoma and CT29 colon cancer cell lines, in which all tested hrRNASET2 regimens inhibited subcutaneous colon cancer and melanoma tumor growth and reduced tumor blood vessel counts and areas, inhibiting melanoma cancer cell metastatic spread to the lung and significantly extending survival times. Moreover, hrRNASET2 treatment activated apoptotic events, which correlated with large lesions in tumor masses, which were wholly absent in Avastin-treated tumors. This observation, which was only detected on histological examination of cross-sections of the excised tumor, demonstrated that tumor mass was actually much lower than the volume determined by palpation. Defects in apoptotic mechanisms play important roles in tumor pathogenesis, allowing neoplastic cells to survive and the tumor mass to expand. In parallel, a wide variety of genetic alterations accumulate, affecting cell proliferation and cell differentiation, and promoting angiogenesis, and tumor invasiveness [14]. We demonstrate herein that hrRNASET2 affects apoptosis and tumor-related alterations due to an anomaly in apoptosis. Moreover, the observed cavities are reflective of hrRNASET2 activity within the tumors, where it destroys existing blood vessels within the tumors, in sharp contrast to other therapeutic agents acting on the peripheral cells of the tumor mass.

Immunostained tumor sections uncovered a distinct tumor cell-free region encompassing the blood vessels, suggested to arise from the immediate effects of infiltration of intravenously administered hrRNASET2 into the tumor tissue. Its direct impact on cells proximal to the endothelium, coupled with its inhibitory influence on cell motility, yielded a distinct tumor cell-free zone, with immediate implications on metastatic spread potential.

These findings lie in agreement with the antitumorigenic and antiangiogenic activities previously reported for the fungal RNASET2 homologue, ACTIBIND, demonstrated to be mediated by its direct competition with the cell proliferation-and angiogenesis-promoting angiogenin. Consequential effects on expression and activity of the matrix metalloproteinase 2 in melanoma and vascular endothelial cells were observed, resulting in attenuated vascularization, alongside heightened incidence of tumor cell apoptosis. Its observed effect on MMP-2, can be of particular relevance in the context of melanoma, notorious for its highly aggressive and angiogenic nature, with ten-year survival rates as low as 10-15% in cases of advanced stage tumors [20].

In conclusion, we surmise that the combined antitumorigenic and antiangiogenic effect of hrRNASET2 results from its direct interaction with the intercellular and extracellular matrices, which lie at the forefront of cell growth, communication and motility processes. Moreover, its similarity to the naturally occurring human protein, renders it safe and nontoxic, as demonstrated in single-dose, repeat-dose toxicity assays. In addition, its selective impact on migrating cells, with no effect on cell proliferation, is expected to dictate a favorable safety profile for human use, with direct consequences on patient quality of life. Moreover, its safety profile will allow for chronic treatment to ensure both treatment success and inhibition of disease recurrence. Due to its marked efficacy, its integration in combination therapies will allow for reduced doses of chemotherapeutic agents. In summary, hrRNASET2 may be an effective anticancer agent, particularly in cases of highly aggressive and mobile tumors.

## MATERIALS AND METHODS

### Recombinant human RNASET2 and CHO-K1 culturing

hrRNASET2 was designed to contain the human growth hormone receptor signal peptide (accession number P10912), a 6xHis-Tag, a spacer region and a TEV protease cleavage site (Supplementary Figure S1). The construct c-DNA was purchased from GeneArt (Regensburg, Germany), and then subcloned into pCDNA3.1(−) (Invitrogene Grand Island, NY, USA).

Chinese hamster ovary (CHO-K1; CCL-61; *Cricetulus griseus*) cells were purchased from the American Type Culture Collection (ATCC; Manassas, VA, USA) and maintained in Dulbecco's modified Eagle Medium (DMEM)/F12 (Sigma, Israel) supplemented with 10% FBS, 100 IU penicillin, 100 μg streptomycin and 0.25 μg amphotericin (BI, Beit Haemek, Israel) at 37°C with 5% CO. Cells were stably transfected with the *hRNASET2* construct using the polyethyleneimine (PEI) method. Transfected cell lines were selected using 400 μg/ml Geneticin Sulphate G418 (Gibco NY, USA) and clonal cell lines were developed by serial dilutions. Single colonies were screened for hrRNASET2 protein expression by an RNase activity assay.

### Isolation of hrRNASET2

Cells were grown to sub-confluence and then transferred to serum-free medium containing F-12 Ham nutrient mixture (Sigma), 100 μg streptomycin and 0.25 μg amphotericin, 2 mM L-glutamine (BI, Beit Haemek, Israel) and BIOGRO-CHO supplement (BI, Beit Haemek, Israel) diluted 1:100. After three days, the growth medium was collected and replaced with fresh medium. The conditioned medium was centrifuged at 4,000 g for 20 min at 4°C, passed through a 0.45 μM filter and stored at −20°C for further use.

The filtrated supernatant was diluted 1:3 with equilibration buffer (20 mM Tris-HCl, pH 8.0; Buffer A) and loaded onto an equilibrated Q-Sepharose FF column (GE Healthcare Bio-Sciences AB, Uppsala, Sweden). The column was washed with Buffer A containing different concentrations (0-25%) of Buffer B (Buffer A + 1M NaCl, pH 8.0). The column was then washed with a 25% to 100% gradient of Buffer B in the equilibration buffer; fractions of 20 ml were collected. RNase activity was determined for each fraction, and the active fractions (between 18% to 25% Buffer B) were pooled. The pool was diluted with buffer C (50 mM Phosphate Buffer, pH 8.0, 500 mM NaCl and 5 mM Imidazol) and loaded onto an equilibrated Hi-Trap-Ni-NTA Sepharose 6 Fast Flow column (GE Healthcare Bio-Sciences AB, Uppsala, Sweden). The column was washed with 2.5 volumes of Buffer C containing 5% and then 10% Buffer D (Buffer C + 300 mM Imidazole), respectively. RNASET2 was then eluted with a gradient step-wise column washings with 10% to 100% Buffer D in Buffer C; fractions of 7 ml were collected. RNase activity and SDS-PAGE were determined for each fraction, and the active fractions were pooled. Intensive dialyses against DDW were performed for 72 h at 4°C. Afterwards, the dialysate was centrifuged at 4,000 x g for 10 min at room temperature and the supernatant was filtered through a 0.22 μM filter.

### RNase activity assay

RNase activity was assessed as described previously [19], with slight modifications. Samples (10 μl) were placed over a plate containing 10 ml 0.1% yeast RNA and 0.8% agarose in 20 mM sodium acetate, pH 4.5, and were incubated (37°C, 30 minutes). Then, the agarose plate was stained with 0.02% (w/v) toluidine blue to detect RNase activity. RNase activity was quantified by placing samples (50 μl) in 50 μl 100 mM sodium acetate buffer (pH 4.5) that contained 4 mg/ml yeast RNA. Blank tubes were prepared in the same way, except that the RNASET2 was replaced with buffer. The reaction mixture was incubated (30 minutes, 50°C); then, 50 μl stop reaction buffer (0.75% w/v uranyl sulfate in 25% weight/volume perchloric acid) were added. After cooling on ice for 5 minutes and centrifugation (15,000 g, 5 min), the supernatant was diluted 20-fold with distilled water, and absorbance was determined at 260 nm in a ND-1000 UV/Vis NanoDrop spectrophotometer (Thermo Fisher Scientific - NanoDrop products, Wilmington, DE, USA).

### SDS-Polyacrylamide Gel-Electrophoresis (SDS-PAGE)

SDS-PAGE was conducted in 12.5% polyacrylamide gel according using the Laemmli procedure [21]. Proteins were stained with Coomassie Blue.

### Western blot analysis

hrRNASET2 (1 μg/well) was loaded onto a SDS-PAGE TGX 4-20% (BioRad Laboratories, Hercules, CA, USA) and transferred onto a Hybond ECL nitrocellulose membrane (GE Healthcare UK Limited, Buckinghamshire, UK). The membrane was blocked overnight at 4°C with 5% skim milk (BioRad Laboratories, Hercules, CA, USA) in Tris-buffered saline (BioRad Laboratories, Munchen, Germany) containing 2% Tween-20 (TBST) under constant shaking, and then washed twice for 10 min with TBST. The membrane was immunolabeled with 1 μg/ml mouse anti-hrRNASET2 (Sigma Aldrich, St Louis, MO, USA), following by goat anti-mouse-alkaline phosphatase IgG (Jackson ImmunoResearch Laboratories, West Grove, PA, USA) at a dilution of 1:10,000. Signals were detected with the Fast/NBT Western blot detection system (Sigma Aldrich, St Louis, MO, USA).

### Binding of RNASET2 to actin in solid phase (ELISA)

hrRNASET2 binding to actin was quantified as modified from Liu *et al*. [22]. Human platelet actin (5 ug/ml) (Cytoskeleton, Denver, CO, USA) was diluted in carbonate-bicarbonate buffer (Sigma-Aldrich, St Louis, MO, USA), pH 9.5, and coated directly onto 96-wells COSTAR EIA/RIA plates (Corning, NY, USA) overnight at 4°C. The plates were then blocked with 5% (w/v) BSA in PBS containing 0.25% Tween-20 (PBST), at room temperature for 1 h, and subsequently incubated with hrRNASET2, at 1:2 serial dilutions in PBST, overnight at 4°C. Plates were washed three times with TBST, under continuous shaking for 10 min each, at room temperature and then incubated with rabbit anti-RNASET2 polyclonal affinity pure antibodies (GENEMED SYNTHESIS, San Antonio, TX, USA), diluted 1:500 in PBST at 37°C for 1 h. After three washing as described above, 0.8 μg/ml peroxidase-conjugated AffiniPure goat anti-rabbit IgG (Jackson ImmunoResearch Laboratories, West Grove, PA, USA) in TBST was added and incubated under the same conditions. Plates were then washed twice with TBST and once with TBS, as described above. 1-Step Ultra TMB-ELISA solution (Thermo Scientific, Pierce Biotechnology, Rockford, IL, USA) was then added and optical absorbance was detected at 650 nm with an Infinite F50 multidetection microplate reader (Tecan, Grödig, Austria).

### Actin-binding assessment by microscale thermophoresis (MST)

The actin-binding capacity (Dissociation Constant, KD) of hrRNASET2 was measured using Monolith NT.115 (Nanotemper Technologies, Germany). Briefly, each sample was tested at serial 1:2 dilutions, from 10 μM to 0.31 μM hrRNASET2 in Buffer G containing ATP. Fluorescently labeled actin, prepared using the MO-L001 Monolith™ Protein Labeling Kit RED-NHS, was then added to each sample. The sample mixtures were incubated for up to 5 min at room temperature and loaded into MST-glass capillaries. Each sample was scanned and measured at 40% IR-Laser Power.

### Human umbilical vein endothelial cell (HUVEC) tube formation assay

HUVEC (CC-2519) were purchased from LONZA (Walkersville, MD USA). HUVEC were maintained in M199 medium supplemented with 20% FCS, 1% glutamine, 1% antibiotic-antimycotic solution, and 50 U/100 ml heparin [19]. The cells were then plated at a density of 14,000 cells/well in a 96-well plate previously coated with growth factor-depleted Matrigel™ (Becton-Dickinson, Bedford, MA) in M199 medium containing 5% FCS and supplemented with angiogenin (R&D Systems Inc., Minneapolis, MN, USA), basic fibroblast growth factor (bFGF, PeproTech Inc., Rocky Hill, NJ, USA) or vascular endothelial growth factor (VEGF, PLR, Rehovot, Israel) at a final concentration of 1 μg/ml each. hrRNASET2 (2 μM final concentration), or Avastin was also added. After overnight incubation at 37°C, the plates were photographed and the extent of tube formation was assessed. Five individual determinations were performed for each treatment.

### Cell invasion assay

Cell invasiveness was carried out using a 24-well BD BioCoatTM Matrigel™ Invasion Chamber (Becton Dickinson Biosciences), according to the manufacturer's directions. Briefly, A375SM cells were grown up to 70 to 80% confluence in MEM, in a 25 mL-flask, released by a brief exposure to trypsin-EDTA, washed and resuspended to 5×10^3^ cells/ml in serum-free medium containing hrRNASET2 at different concentrations (2.5, 1.0 or 0.5 uM) or PBS (control). Then, cells were seeded in the upper compartment of the 24-well plate; the lower compartment was immediately filled with 800 μl NIH 3T3 fibroblast-conditioned medium [14], provided as a chemoattractant. After 24 h incubation at 37°C and 95% O and 5% CO, cells on the upper surface of the membrane were carefully removed and the membranes were stained with Diff-Quick solution. Cells that migrated to the lower surface of the membrane were counted under the microscope (Leica DMI 3000M, Germany) at ×10 magnification. The data are expressed as the average number of cells counted in 10 fields in each of the three experiments ± SD.

### Animal studies

All the *in vivo* animal model experiments were performed with the approval of the Animal Experimental Ethics Committee of the Israel Health Ministry.

### Xenograft model

Viable human colon cancer HT29 cells (1×10^6^/100 μl per mouse) were subcutaneously (s.c.) injected into the left hip of 6–8-week-old female athymic mice (nu/nu; Harlan, Israel), on a normal ad libitum diet and housed under pathogen-free conditions. Two weeks thereafter, when tumors were 80–100 mm^3^, mice were randomized into the following treatment groups (*n=* 6): Group 1: hrRNASET2 (1 mg/kg), Group2: hrRNASET2 (0.5 mg/kg), Group 3: hrRNASET2 (0.05 mg/kg), Group 4: PBS (Control) and Group 5: Avastin (5 mg/kg, as positive control). Treatments were intravenously (i.v.) administered every other day, for 30 days. Tumors were measured with calipers 3 times per week. Tumor size (mm^3^) was evaluated by measuring the two greatest perpendicular dimensions with Vernier calipers and using the formula 1/2LW [23] in which L is the greatest dimension and W is the dimension perpendicular to L; results are presented as mean values. The experiments were terminated 30 days after the initial treatment, when mice were sacrificed, and tumors were extracted for histopathologic examination. Tumors were fixed, and 6 μM paraffin sections were stained with H&E and with the FragEl-DNA Fragmentation Detection kit Colorimetric Klenow Enzyme (Calbiochem, Darmstadt, Germany), according to the supplier’s instructions, to evaluate apoptosis. The number of blood vessels in each median tumor cross section was counted, and their areas were analyzed with Image J (NIH) software. Relative area was calculated as the ratio between the total blood vessel area and tumor section area (n = 6 tumors; 3 microscopic fields for each). The course taken by exogenously administered hrRNASET2 was monitored by staining tumor sections with rabbit anti-RNASET2 (SIGMA Prestige Antibodies St. Louis MO, USA), using the ABC Goat Staining Kit System-ImmunoCruz™ (Santa Cruz Biotechnology, Dallas, TX, USA), following the manufacturer instructions.

### Survival animal studies in xenograft model

Viable human melanoma A375SM cells (2.5×10^5^/100 μl per mouse) were s.c. injected into the left hip of 6-8-week-old age female athymic mice (nu/nu; Harlan, Israel), on a normal ad libitum diet and housed under pathogen-free conditions. Two weeks thereafter, when tumors were 80–100 mm^3^, the mice were randomized into the following treatment groups (n=10): Group 1: hrRNASET2 (1 mg/kg) or Group 2: PBS, (Control), was administered every other day, for 73 days. Group 3: Dacarbazine (DTIC, 80 mg/kg, as positive control) was administered on days 10 to 14 from treatment start and Group 4: hrRNASET2 (1 mg/kg) and DTIC, (80 mg/kg), were injected into the same mouse as per the schedules described for Groups 1 and 3. Tumors were measured with calipers as described above. Euthanization was performed as per the Institutional Animal Care and Use Committee (IACUC) guidelines: upon loss of 10% or more of body weight, or when tumor volume reached 1.5 cm diameter, or when the mouse showed any clinical sign indicative of morbidity. Results are presented as the median survival days (including range) as determined from a Kaplan–Meier plot.

### Experimental lung metastasis model

Viable human melanoma A375SM cells (1×10^6^/100 μl per mouse) were i.v injected into the lateral vein of 6–8-week-old female athymic mice (nu/nu; Harlan, Israel), on a normal ad libitum diet and housed under pathogen-free conditions. Five days thereafter, animal groups (n=10) were treated as follows: Group 1: hrRNASET2 (1 mg/kg) and Group2: PBS (Control) were treated every other day, for 60 days. Group 3: hrRNASET2 (1 mg/kg) was treated once a week, for 60 days and Group 4: Avastin (2.5 mg/kg, as positive control) was treated twice a week, for 60 days. The experiments were terminated 60 days after the initial treatment, all mice were sacrificed, autopsied, and lungs were fixed with Bouin's solution. The number of macroscopic tumor nodules was counted under a dissecting microscope.

## SUPPLEMENTARY MATERIALS FIGURES



## Abbreviations

bFGF: basic fibroblast growth factor
BSA: bovine serum albumin
CHO: Chinese hamster ovary
DMEM: Dulbecco’s Modified Eagle’s Medium
EDC: 1-[3-(dimethylamino)-propyl]-3-ethyl-carboimide methiodide
EDTA: ethylenediaminetetraacetic acid
EK: enterokinase
FBS: fetal bovine serum
FCS: fetal calf serum
H&E: hematoxylin and eosin
hrRNASET2: recombinant human RNASET2
HUVEC: human umbilical vein endothelial cell
IPTG: isopropyl β-D-thiogalactopyranoside
MMP2: matrix metalloprotein 2
PBS: phosphate buffered saline
RNASE: ribonuclease
RTV: relative tumor volume
SAB: sample application buffer
SDS-PAGE: sodium dodecyl sulfate polyacrylamide gel electrophoresis
TBS: tris buffer saline
TBST: tris buffer saline with tween-20
TEV: tobacco Etch virus
VEGF: vascular endothelial growth factor
