# Supplementary material for: Human recombinant RNASET2: A potential anti-cancer drug
Source: Oncoscience. 2016 Mar 4;3(2):71–84. doi: 10.18632/oncoscience.295 (PMC4789573; doi:10.18632/oncoscience.295)
Supplement: Supplementary file 1 [file oncoscience-03-71-s001.pdf]

SUPPLEMENTARY FIGURES

```
                                                    BstAP
          Sac1           Not1                       AlwN1
        Ecl2 |    EcoRV  Eag1     Nco1            Pvu2  |
           | |        |     |        |               |  |
        CGAGCTCGGATCGATATCTGCGGCCGCCACCATGGACCTGTGGCAGCTGCTGCTGACCCT
     1  ---------+---------+---------+---------+---------+---------+ 60
        GCTCGAGCCTAGCTATAGACGCCGGCGGTGGTACCTGGACACCGTCGACGACGACTGGGA
                                       M  D  L  W  Q  L  L  L  T  L

              Nae1
           NgoM4 |
       BseR1   | |
           |   | |
        GGCCCTGGCCGGCTCCTCTGACGCCCACCACCACCATCACCACGACTACGACATCCCTAC
    61  ---------+---------+---------+---------+---------+---------+ 120
        CCGGGACCGGCCGAGGAGACTGCGGGTGGTGGTGGTAGTGGTGCTGATGCTGTAGGGATG
         A  L  A  G  S  S  D  A  H  H  H  H  H  H  D  Y  D  I  P  T

                                                  BssS1
                                                      |
        CACCGAGAACCTGTACTTTCAGGGCGACAAGCGGCTGCGGGACAACCACGAGTGGAAAAA
   121  ---------+---------+---------+---------+---------+---------+ 180
        GTGGCTCTTGGACATGAAAGTCCCGCTGTTCGCCGACGCCCTGTTGGTGCTCACCTTTTT
         T  E  N  L  Y  F  Q  G  D  K  R  L  R  D  N  H  E  W  K  K

        ACTGATCATGGTCCAGCACTGGCCTGAGACAGTGTGCGAGAAGATCCAGAACGACTGCCG
   181  ---------+---------+---------+---------+---------+---------+ 240
        TGACTAGTACCAGGTCGTGACCGGACTCTGTCACACGCTCTTCTAGGTCTTGCTGACGGC
         L  I  M  V  Q  H  W  P  E  T  V  C  E  K  I  Q  N  D  C  R

    SanD1
        |
        GGACCCTCCTGACTACTGGACCATCCACGGCCTGTGGCCTGACAAGTCCGAGGGCTGCAA
   241  ---------+---------+---------+---------+---------+---------+ 300
        CCTGGGAGGACTGATGACCTGGTAGGTGCCGGACACCGGACTGTTCAGGCTCCCGACGTT
         D  P  P  D  Y  W  T  I  H  G  L  W  P  D  K  S  E  G  C  N

    Age1              SpAcc
       |                  |
        CCGGTCCTGGCCTTTCAACCTGGAAGAAATCAAGGATCTGCTGCCTGAGATGCGGGCCTA
   301  ---------+---------+---------+---------+---------+---------+ 360
        GGCCAGGACCGGAAAGTTGGACCTTCTTTAGTTCCTAGACGACGGACTCTACGCCCGGAT
         R  S  W  P  F  N  L  E  E  I  K  D  L  L  P  E  M  R  A  Y

     BmgB1                Age1
                 |                   |
        CTGGCCTGACGTGATCCACTCCTTCCCTAACCGGTCCCGGTTCTGGAAGCATGAGTGGGA
   361  ---------+---------+---------+---------+---------+---------+ 420
        GACCGGACTGCACTAGGTGAGGAAGGGATTGGCCAGGGCCAAGACCTTCGTACTCACCCT
         W  P  D  V  I  H  S  F  P  N  R  S  R  F  W  K  H  E  W  E
```

(Continued)

```
                                                       Xmn1
                            Sal1                       Sca1 Eag1
                               |                          |    |
      GAAGCACGGCACTTGTGCTGCCCAGGTCGACGCCCTGAACTCCCAGAAGAAGTACTTCGG
421   ---------+---------+---------+---------+---------+---------+ 480
      CTTCGTGCCGTGAACACGACGGGTCCAGCTGCGGGACTTGAGGGTCTTCTTCATGAAGCC
       K  H  G  T  C  A  A  Q  V  D  A  L  N  S  Q  K  K  Y  F  G

                                                     BseY1
                     BsaXb                         BsaXa |
                         |                             | |
      CCGGTCCCTGGAACTGTACCGGGAGCTGGACCTGAACTCCGTGCTGCTGAAGCTGGGCAT
481   ---------+---------+---------+---------+---------+---------+ 540
      GGCCAGGGACCTTGACATGGCCCTCGACCTGGACTTGAGGCACGACGACTTCGACCCGTA
       R  S  L  E  L  Y  R  E  L  D  L  N  S  V  L  L  K  L  G  I

         Eco57                                    Sma1
             |                                            |
      CAAGCCTTCCATCAACTACTACCAGGTGGCCGACTTCAAGGACGCCCTGGCCCGGGTGTA
541   ---------+---------+---------+---------+---------+---------+ 600
      GTTCGGAAGGTAGTTGATGATGGTCCACCGGCTGAAGTTCCTGCGGGACCGGGCCCACAT
       K  P  S  I  N  Y  Y  Q  V  A  D  F  K  D  A  L  A  R  V  Y

                          AlwN1          SpAcc
                              |              |
      CGGCGTGATCCCTAAGATCCAGTGCCTGCCTCCTTCCCAGGACGAAGAAGTGCAGACCAT
601   ---------+---------+---------+---------+---------+---------+ 660
      GCCGCACTAGGGATTCTAGGTCACGGACGGAGGAAGGGTCCTGCTTCTTCACGTCTGGTA
       G  V  I  P  K  I  Q  C  L  P  P  S  Q  D  E  E  V  Q  T  I
                                Pvu2
             Bsg1                   Bsg1 AlwN1 Pst1
                |                      |     |    |
      CGGCCAGATCGAGCTGTGCCTGACCAAGCAGGACCAGCAGCTGCAGAACTGCACCGAGCC
661   ---------+---------+---------+---------+---------+---------+ 720
      GCCGGTCTAGCTCGACACGGACTGGTTCGTCCTGGTCGTCGACGTCTTGACGTGGCTCGG
       G  Q  I  E  L  C  L  T  K  Q  D  Q  Q  L  Q  N  C  T  E  P
   Bpu10                Msc1
                       |                   |
      TGGCGAGCAGCCTTCCCCTAAGCAGGAAGTCTGGCTGGCCAACGGCGCTGCCGAGTCCAG
721   ---------+---------+---------+---------+---------+---------+ 780
      ACCGCTCGTCGGAAGGGGATTCGTCCTTCAGACCGACCGGTTGCCGCGACGGCTCAGGTC
       G  E  Q  P  S  P  K  Q  E  V  W  L  A  N  G  A  A  E  S  R

     Stu1                      BseR1
        |                          |
      AGGCCTGCGCGTGTGCGAGGACGGCCCTGTGTTCTACCCTCCTCCCAAGAAAACCAAGCA
781   ---------+---------+---------+---------+---------+---------+ 840
      TCCGGACGCGCACACGCTCCTGCCGGGACACAAGATGGGAGGAGGGTTCTTTTGGTTCGT
       G  L  R  V  C  E  D  G  P  V  F  Y  P  P  P  K  K  T  K  H

      EcoR1    BamH1  Spe1    Eag1        BstX1
          |        |     |       |            |
      CTAGGAATTCAGTGGATCCACTAGTAACGGCCGCCAGTGTGCTGGAA
841   ---------+---------+---------+---------+------- 887
      GATCCTTAAGTCACCTAGGTGATCATTGCCGGCGGTCACACGACCTT
       *
```

Supplementary Figure S1 (*Continued*): Restriction map. The human recombinant RNASET2 construct. The amino acid sequence is in bold. The human growth hormone receptor signal peptide (magenta), 6 x His tag (green), spacer region (blue) and TEV protease cleavage site (grey) were situated upstream to the mature RNASET2 sequence. The c-DNA sequence was optimized for expression in CHO. The open reading frame ends with a stop codon (asterisk).

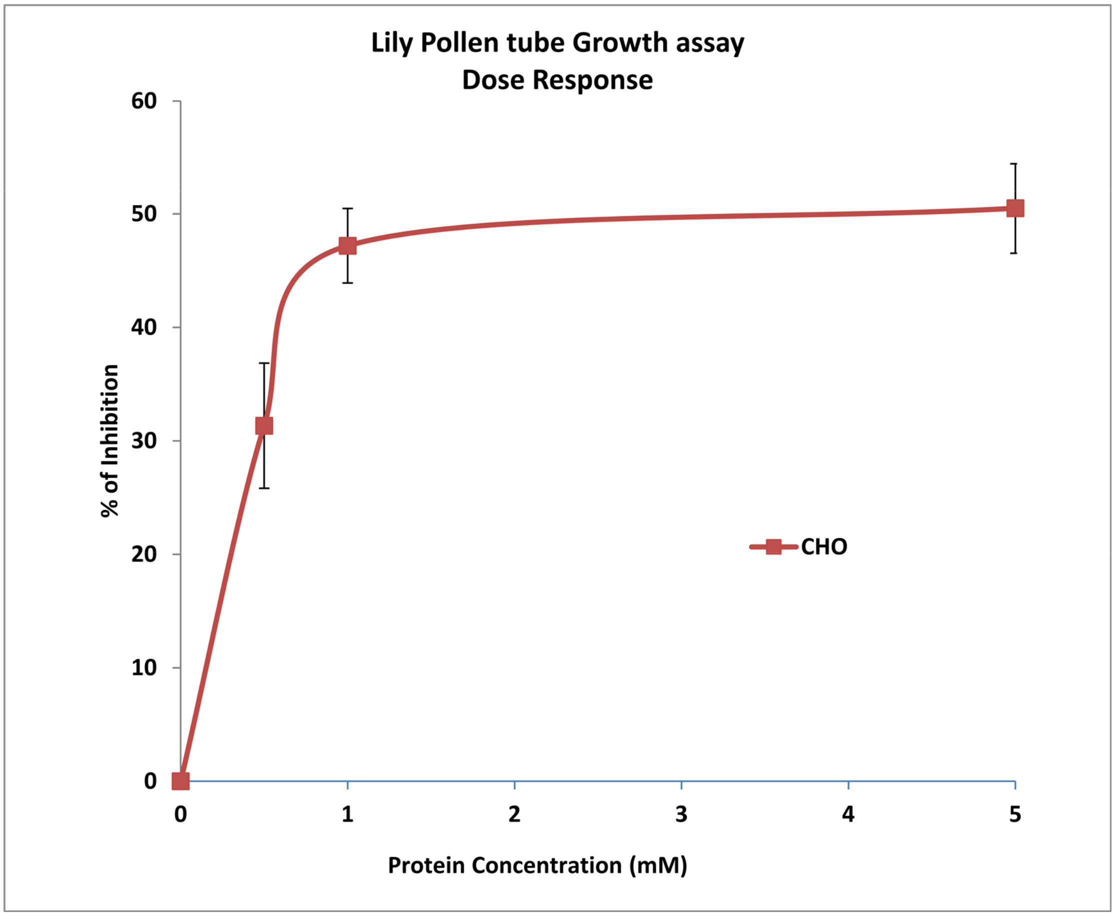

Supplementary Figure S2: Lily pollen tube results.

Lilly pollen tube growth (PTG) assay

Pollen tube growth was assessed as previously described (4), with slight modifications. Lilium longiflorum var. white heaven flowers were allowed to open in the lab. Anthers were excised and brought to complete dehiscence and pollen release for 24-48 h at room temperature. The pollen was suspended at a concentration of about 40 mg per ml culture medium (7% sucrose (w/v), 1.27 mM $Ca(NO_3)_2$, 0.16 mM H_2BO_3, 1 mM KNO_3 and 3 mM KH_2PO_4 in water) (5), vigorously stirred by vortex and centrifuged for 2 min at 13,400 rpm at room temperature, to release the lipid phase from pollen grains. The cell pellet was suspended in 1 ml culture medium, in a fresh 1.5 ml Eppendorff tube. The same procedure was repeated three more times. Cells were counted and suspended in culture medium at a density of 25,000 cells/ml. Pollen was germinated in 100 µl culture medium containing 1 mM hrRNASET2 or PBS (as control) and incubated for 1-2 h at 25°C in the dark, to maximum 500 mm. The pollen tubes were fixed and stained by adding 100 ml Alexander stain (20 mg malachite green, 100 mg acid fuchsin, 5 gr phenol, 2 ml lactic acid, 20 ml ethanol, 40 ml glycerol, 50 ml water) (6) to each tube and gently mixing. Pollen tube length were imaged under a microscope (Leica DMi 3000M, Germany) equipped with a Digital Sight DS-Fi1 camera (Nikon, Japan) and analyzed using NIS-Elements Br software (Nikon, Japan). N=3 and at least 100 pollen tubes were measured in each determination.
